# Limited sucrose intake produces subregion-specific remodeling of perineuronal nets in the medial prefrontal cortex

**DOI:** 10.1007/s00429-026-03141-5

**Published:** 2026-07-15

**Authors:** Houda Nashawi, Corey T. Foltz, Nakyung Oh, Eve A. Santiago, Dana R. Selm, James P. Herman, Yvonne M. Ulrich-Lai

**Affiliations:** 1 Department of Pharmacology, Physiology, and Neurobiology, University of Cincinnati, Reading Campus, 2120 E Galbraith Rd—ML 0512, Cincinnati, OH 45237 USA; 2 Neuroscience Graduate Program, University of Cincinnati, Cincinnati, OH USA

**Keywords:** Sucrose, Stress, Parvalbumin, Perineuronal nets, Medial prefrontal cortex, Structural plasticity

## Abstract

Despite growing recognition that natural rewards can mitigate stress responses, the underlying neurobiological mechanisms remain poorly understood. Here, we examined whether limited sucrose intake (LSI), a paradigm of repeated natural food reward, induces structural plasticity in parvalbumin-expressing (PV+) interneurons and their perineuronal nets (PNNs) within the medial prefrontal cortex (mPFC), a key region involved in stress and reward regulation. In a 2 × 2 design, adult male rats were exposed to 14 days of LSI (vs. water), along with repeated restraint stress (vs. no stress). Immunohistochemical analyses were used to assess PV+ interneurons, PNNs, and glutamatergic and GABAergic perisomatic appositions onto PV+ neurons in the prelimbic (PL) and infralimbic (IL) prefrontal cortices. While repeated restraint stress produced minimal effects, LSI induced subregion-specific changes. In the PL, LSI increased PNN investment on PV+ cells and reduced both excitatory and inhibitory appositions without altering their ratio. In contrast, in the IL, LSI shifted PNNs from full to partial forms without changing their overall prevalence. This was associated with an increased excitatory/inhibitory apposition ratio that was restricted to PNN-negative cells in stress-naïve rats. While the functional consequences remain to be established, these LSI-induced PNN-mediated changes may serve to stabilize PV+ interneuron function and inform hypotheses regarding how natural rewards modulate prefrontal microcircuits relevant to stress regulation.

**Supplementary Information:**

The online version contains supplementary material available at https://doi.org/10.1007/s00429-026-03141-5.

## Introduction

Neuronal circuits in the mammalian brain exhibit remarkable plasticity, enabling adaptation to environmental changes and the maintenance of cognitive and emotional function throughout life (Kays et al. 2012; Kolb and Gibb 2014). While synaptic mechanisms of plasticity have been extensively studied, the role of the extracellular matrix (ECM) in modulating neuronal function and plasticity is increasingly recognized (Dityatev and Schachner 2003; Barros et al. 2011; Frischknecht and Gundelfinger 2012; Fawcett et al. 2019). Within the ECM, perineuronal nets (PNNs) are lattice-like structures primarily composed of chondroitin sulfate proteoglycans (CSPGs) (Kwok et al. 2010, 2011; Giamanco and Matthews 2012; Oohashi et al. 2015) that preferentially enwrap the cell bodies and proximal dendrites of fast-spiking parvalbumin-expressing (PV+) interneurons (Testa et al. 2019; Carceller et al. 2023), providing structural support, neuroprotection, and regulation of synaptic activity and plasticity (Sorg et al. 2016; Fawcett et al. 2019; Bosiacki et al. 2019). Notably, PNNs are highly dynamic and responsive to environmental stimuli, allowing them to stabilize or remodel neuronal circuits in response to changing demands (Reichelt et al. 2019b; Santos-Silva et al. 2024; Sanchez et al. 2024). This capacity for experience-dependent plasticity suggests that PNNs may contribute to the brain’s ability to adapt and recover from environmental challenges, a process broadly referred to as stress resilience (Herrman et al. 2011; Karatsoreos and McEwen 2011; Russo et al. 2012; Scheffer et al. 2018; Spencer-Segal and Akil 2019; Cathomas et al. 2019).

Stress is often defined as real or perceived threats to homeostasis or well-being that evoke acute, adaptive physiological and behavioral responses (Ulrich-Lai and Herman 2009; Chrousos 2009; Godoy et al. 2018). However, when these responses are prolonged or repeatedly activated, they can disrupt neural function and contribute to physical and mental health disorders (Schneiderman et al. 2005; Chrousos 2009; Godoy et al. 2018). Importantly, not all individuals exposed to stress experience adverse outcomes to the same extent. Growing evidence suggests that regular engagement in rewarding activities, such as enjoying physical exercise, time in nature, creative hobbies, or comfort foods, can enhance resilience to the effects of stress on mood and health (Pressman et al. 2009; Zawadzki et al. 2015; Fancourt et al. 2021; Dutcher 2023). In fact, such activities are increasingly being incorporated into therapeutic interventions for stress-related disorders (Keefe et al. 1996; Dimidjian et al. 2006; Garland 2016), where they are found to enhance treatment outcomes through positive environmental stimulation. Despite their evident effectiveness, the mechanisms by which natural rewards confer their stress-buffering effects remain unclear.

To address this, our laboratory developed a rat model of natural reward using limited sucrose intake (LSI), leveraging rats’ preference for sweet substances (Sclafani 1991). In this paradigm, rats with free access to standard chow and water are given brief, twice-daily access to a small amount (4 mL) of 30% sucrose solution. Within two weeks, LSI attenuates hypothalamic-pituitary-adrenocortical (HPA) axis and sympathetic responses to acute stress, shown by reduced plasma corticosterone levels and tachycardia, respectively (Ulrich-Lai et al. 2007, 2010, 2011; Christiansen et al. 2011; Packard et al. 2017; Egan et al. 2019; Almehmadi et al. 2023). LSI also decreases stress-related behaviors, with reduced struggling during restraint (Egan et al. 2019) and increased sociability and exploration in the social interaction and elevated plus-maze tests (Ulrich-Lai et al. 2010; Egan et al. 2019).

The medial prefrontal cortex (mPFC) plays a central role in stress response regulation. Through its extensive connectivity with limbic, hypothalamic, and brainstem structures, it coordinates behavioral, neuroendocrine, and autonomic stress responses and modulates emotional and cognitive responses to stressors (Herman and Cullinan 1997; Herman et al. 2005a; Ulrich-Lai and Herman 2009; McEwen and Morrison 2013; McKlveen et al. 2015). The mPFC is also a core component of the mesocorticolimbic reward system (Tzschentke 2000; Wise 2000; Haber and Knutson 2010; Russo and Nestler 2013; Pastor and Medina 2021) and is responsive to palatable food consumption (Mendoza et al. 2005; Petykó et al. 2009). This convergence of stress- and reward-related functions positions the mPFC to mediate the stress-blunting effects of LSI. Supporting this idea, the mPFC emerged in both correlational and Bayesian network analyses as a key node whose contribution to stress regulation is likely altered by LSI (Ulrich-Lai et al. 2016). LSI also alters mPFC functional connectivity with other stress-regulatory regions, including the basolateral amygdala (BLA), which receives abundant mPFC input and is required for LSI-induced stress blunting (Coover et al. 1973; Feldman et al. 1983; Szafarczyk et al. 1986; Mcdonald et al. 1996, 1999; Goldstein et al. 1996; McDonald 1998; Vertes 2004; Bhatnagar et al. 2004; Gabbott et al. 2005; Ulrich-Lai et al. 2010; Pinard et al. 2012; Hübner et al. 2014).

One mechanism that may underlie this reorganization is plasticity within local inhibitory microcircuits. PV+ interneurons, which constitute the majority of mPFC interneurons (Markram et al. 2004; McKlveen et al. 2015; Bittar and Labonté 2021), form perisomatic synapses onto pyramidal neurons, providing powerful inhibition that regulates excitability and balances network activity (Mueller-Buehl et al. 2023). Given that PNNs are major regulators of PV+ interneuron activity and plasticity and are sensitive to environmental stimuli (Reichelt et al. 2019b; Santos-Silva et al. 2024; Sanchez et al. 2024), their remodeling may underlie LSI-induced plasticity and functional shifts observed in the mPFC. Our previous work showed that LSI enhances PNN integrity and alters vGLUT1 + and vGAT+ appositions onto PV+ interneurons in the BLA (Nashawi et al. 2025), suggesting a mechanism for reward-driven stress resilience. Whether similar plasticity occurs in the mPFC is unknown. Here, we investigate how LSI and repeated restraint stress affect PNNs and vGLUT1/vGAT appositions onto PV+ interneurons in the prelimbic (PL) and infralimbic (IL) subregions of the mPFC, aiming to reveal microcircuit changes that may contribute to the resilience-promoting effects of natural rewards.

## Materials and methods

### Animals and experimental design

To minimize animal use, tissue for this study was obtained from animals used in experiments previously described in (Nashawi et al. 2025). Briefly, male Long-Evans rats (*n* = 48; 250–275 g) were obtained from Envigo (Indianapolis, IN, USA). They were singly housed in a temperature- and humidity-controlled facility on a 12-hour light/dark cycle (lights on at 06:00 h and off at 18:00 h), with food (standard rodent chow; Teklad LM-485; Envigo, Madison, WI, USA) and water available *ad libitum*. Housing facilities are accredited by the Association for the Assessment and Accreditation of Laboratory Animal Care (AAALAC).

**Fig. 1:**
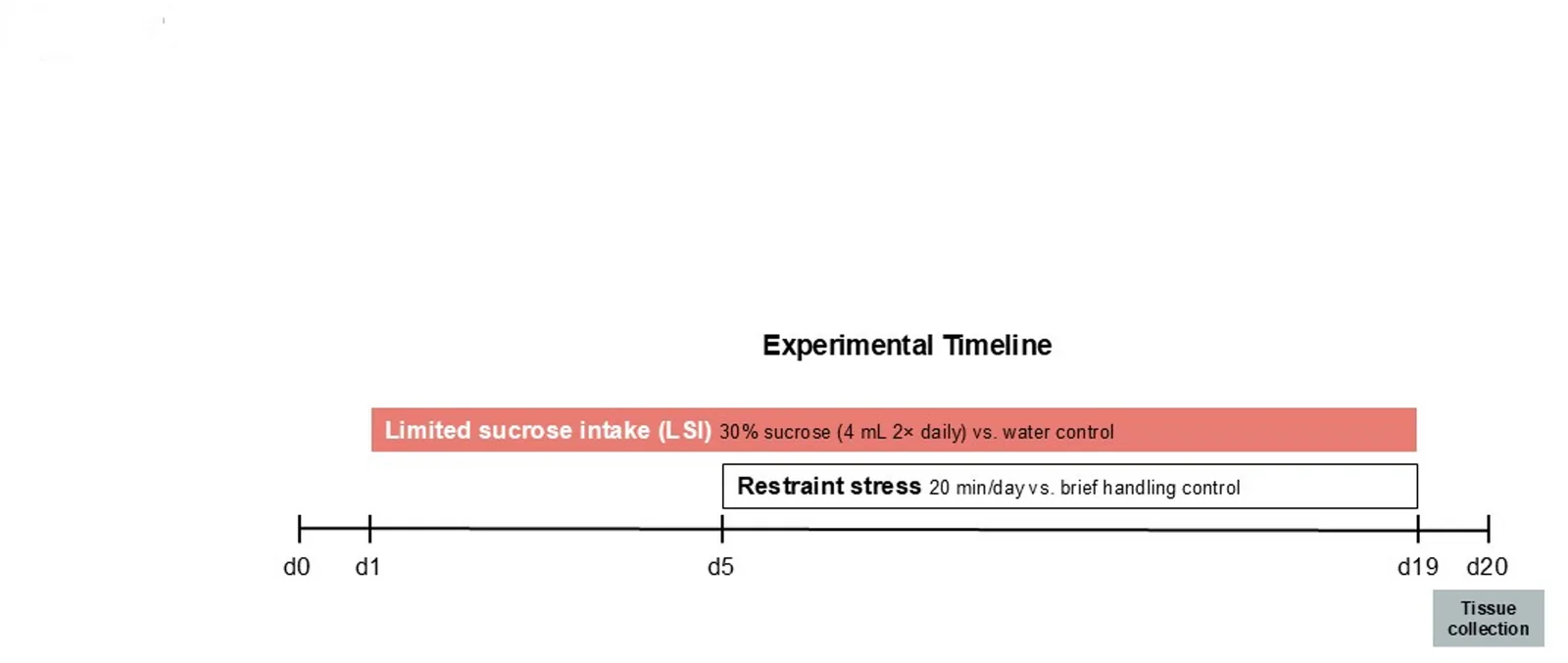
Experimental timeline. Limited sucrose intake (LSI) began on d1, in which rats with ad libitum access to normal chow and water were given additional access to up to 4 ml of a 30% sucrose solution (vs. water as a control) twice daily. Restraint stress was introduced on d5 and continued for 20 min per day for 15 days, while non-stressed animals were briefly handled without restraint. On d20, animals were euthanized, and brain tissue was collected for immunohistochemical analyses of PV+ cells, PNNs, and perisomatic appositions in the prelimbic (PL) and infralimbic (IL) medial prefrontal cortex

After a one-week acclimation period, rats were randomly assigned to one of four experimental groups (matched for initial body weight) in a 2 × 2 design (*n* = 12 rats/group), with factors of stress (repeated restraint stress vs. no stress control) and sucrose intake (LSI vs. water control). LSI began on day 1 and continued for 19 days. Starting on day 5, rats in the stress groups underwent daily restraint for 14 days. Body weight and food intake were monitored periodically. On day 20, animals were euthanized and brains collected for immunohistochemistry (Fig. 1). All procedures were approved by the Institutional Animal Care and Use Committee (IACUC) of the University of Cincinnati and adhered to the United States National Institutes of Health (NIH) Guide for the Care and Use of Laboratory Animals.

### Limited sucrose intake (LSI) protocol

The LSI protocol was carried out as described previously (Ulrich-Lai et al. 2007, 2010, 2011, 2016; Christiansen et al. 2011; Egan and Ulrich-Lai 2015; Packard et al. 2017; Egan et al. 2018, 2019; Almehmadi et al. 2023; Nashawi et al. 2025). In brief, all rats had free access to normal chow (Teklad LM-485; Envigo, Madison, WI, USA) and a homecage water bottle at all times. Rats in the sucrose groups were additionally provided a limited amount (4 mL) of 30% sucrose solution (821721; MP Biomedicals, Solon, OH) twice daily (at 09:30 h and 16:00 h). The sucrose access session for each rat ended either when the rat had consumed the entire 4 mL of solution or after 30 min, whichever occurred first. Control rats, which received twice-daily access to 4 ml of water instead of sucrose solution, typically did not consume the full amount; therefore, their session duration was matched to that of the sucrose-fed rats. The volume of drink consumed by each rat was recorded at the end of each session.

### Repeated restraint stress protocol

Starting on experimental day 5, rats assigned to the stress groups underwent restraint stress for 20 min per day over 14 consecutive days. Restraint was performed during the light phase (at 11:30 h) using well-ventilated acrylic restraint tubes. Control rats were handled daily for ~ 1 min at the same time of day but were not restrained. Restraint stress was chosen for this experiment as a homotypic stress paradigm to facilitate comparisons with our homotypic reward paradigm (i.e., LSI).

### Tissue processing

On d20, rats were deeply anesthetized with pentobarbital and transcardially perfused, first with normal saline then with 3.7% paraformaldehyde (PFA; Fisher Scientific, Fair Lawn, NJ, USA) in 1X phosphate buffered-saline (PBS). Brains were extracted, post-fixed in 3.7% PFA at room temperature overnight, then transferred to a 30% sucrose solution in 1X PBS at 4 °C until they sank. Coronal Sect.  (30 μm) containing the PL and IL regions of the prefrontal cortex were collected using a freezing microtome (SM2010; Leica Microsystems, Wetzlar, Germany) and were stored at -20 °C in cryoprotectant (30% sucrose, 1% polyvinylpyrrolidone-40, and 30% ethylene glycol in 1X PBS) until use.

### Immunohistochemistry

Free-floating sections were washed 5 times (5 min per wash) in 1X PBS and then blocked in 0.1% bovine serum albumin (BSA; A4503; Sigma-Aldrich) and 0.4% Triton X-100 (93443; Sigma-Aldrich) in 1X PBS for 1 h at room temperature. Sections were then incubated overnight at 4 °C in blocking solution containing biotin-conjugated *Wisteria floribunda* agglutinin (WFA; L1516; Sigma-Aldrich; 1:1000) to label PNNs (Härtig et al. 1992; Brückner et al. 1996) and primary antibodies against PV (mouse monoclonal; P3088; Sigma-Aldrich; 1:2000) and either vGLUT1 (rabbit polyclonal; 135303; Synaptic Systems; 1:1000) or vGAT (rabbit polyclonal; 131002; Synaptic Systems, Gottingen, Germany; 1:1000) to label PV+ interneurons and glutamatergic or GABAergic terminals, respectively. After washing in 1X PBS (5 × 5 min), sections were incubated overnight at 4 °C in blocking solution containing Alexa Fluor™ 488-conjugated streptavidin (S11223; ThermoFisher Scientific; Walkham, MA, USA; 1:500), cyanine5-conjugated goat anti-mouse antibody (A10524; ThermoFisher Scientific; 1:500), and cyanine3-conjugated goat anti-rabbit antibody (A10520; ThermoFisher Scientific; 1:500) in the dark. Sections were finally washed in 1X PBS (5 times × 5 min), mounted on glass slides (Gold Seal Products; Portsmouth, NH, USA), and cover-slipped with polyvinyl alcohol mounting medium with DABCO^®^ (10981; Sigma-Aldrich).

### Confocal microscopy and analysis

All images were captured using a Nikon C2 laser-scanning confocal microscope (Nikon Instruments Inc, Tokyo, Japan). For PV+ interneuron and PNN quantification, a 20× objective was used to capture multichannel Z-stacks (~ 40 optical sections; 0.4 μm step size) bilaterally from two levels of the PL and IL cortical regions at approximately 3.24 and 3.00 mm from bregma, yielding 4 images per rat, whenever feasible. PV+ cells were classified as having full PNNs (continuous WFA labeling surrounding the entire soma and extending onto proximal neurites), partial PNNs (incomplete somatic coverage and/or lack of extension onto neurites), or no PNNs (see Fig. 3 for examples). Full PNNs represent more complete and structurally mature extracellular matrix envelopment, whereas partial PNNs likely reflect intermediate or actively remodeling states. Distinguishing these categories allows the detection of subtle, experience‑dependent ECM reorganization that might be missed when only PNN presence/absence is scored. The total number of PV+ cells in each category was manually counted within predefined PL and IL cortical boundaries, determined using the Paxinos and Watson brain atlas (Paxinos and Watson 2009). Quantification was performed using the multi-point tool in ImageJ (NIH, Bethesda, MD). The proportion of PV+ cells in each category was calculated relative to the total number of PV+ cells per image.

To visualize perisomatic puncta expressing vGLUT1 and vGAT in apposition to PV+ interneurons, multichannel z-stack images (~ 30 optical sections; 0.2 μm step size) were acquired bilaterally using a water-immersion 60× objective lens from two anatomical levels within the PL and IL cortices, corresponding to those used for PV+ cell and PNN quantification. To evaluate the potential impact of PNN investment on synaptic inputs, 4 PV+ cells with PNNs and 4 without were randomly selected per image for each animal, as previously described (Smail et al. 2023; Nashawi et al. 2025). The central focal plane of each cell was identified, and its perimeter was delineated using the freehand selection tool in ImageJ. Subsequently, vGLUT1 + and vGAT+ puncta within 1 μm of the PV+ soma were counted across the three centermost optical sections using the multipoint tool in ImageJ, and were then averaged to yield the mean perisomatic vGLUT1 and vGAT puncta density per PV+ cell. The 1 μm threshold was selected to maintain consistency with prior work from our group (Smail et al. 2023; Nashawi et al. 2025). Puncta number and fluorescence intensity were analyzed as complementary measures of presynaptic organization. Puncta number is used as a proxy for the density of putative appositions onto PV+ cells, while fluorescence intensity is used as a proxy for presynaptic protein content within those puncta, which may change independently from apposition number. Additionally, PV+ soma size (defined by cell perimeter) and PV, WFA, vGLUT1, and vGAT fluorescence intensities (defined as integrated density) were quantified. All analyses were performed by an investigator blinded to experimental conditions to minimize potential bias. For all cell-level measures (including perisomatic puncta, fluorescence intensity, and PV+ cell size), values from PV+ cells were averaged within each animal prior to statistical analysis (i.e., 4 PV+ cells with PNNs and 4 without per animal). Statistical comparisons were then performed using these animal-level means, such that the animal served as the unit of analysis. For comparisons between PV+ cells with and without PNNs, paired analyses were conducted within the same animal to account for within-subject variability.

### Statistical analysis

Data were analyzed using GraphPad Prism 9. Two-way analysis of variance (ANOVA) was used to assess the effects of sucrose consumption (DRINK: water vs. sucrose) and stress exposure (STRESS: no stress vs. stress) on PV+ cell counts, PNN expression, and perisomatic puncta apposing PV+ cells. Three-way ANOVAs (DRINK × STRESS × PNN PRESENCE) were conducted to determine whether treatment effects on perisomatic puncta were influenced by PNN presence, which was included as a repeated measures factor. Paired two-tailed t-tests were used to compare parameters between PV+ cells with and without PNNs within the same rats. Datapoints were excluded as outliers only if they exceeded 1.96 standard deviations from the mean and were beyond 1.5 times the interquartile range from the lower or upper quartile (Ulrich-Lai et al. 2007, 2010, 2011; Christiansen et al. 2011; Egan and Ulrich-Lai 2015; Egan et al. 2018, 2019; Almehmadi et al. 2023). Statistical significance was set at *p* < 0.05. All data are presented as mean ± SEM.

## Results

### Effect of LSI and repeated restraint stress on food intake and body weight

**Fig. 2:**
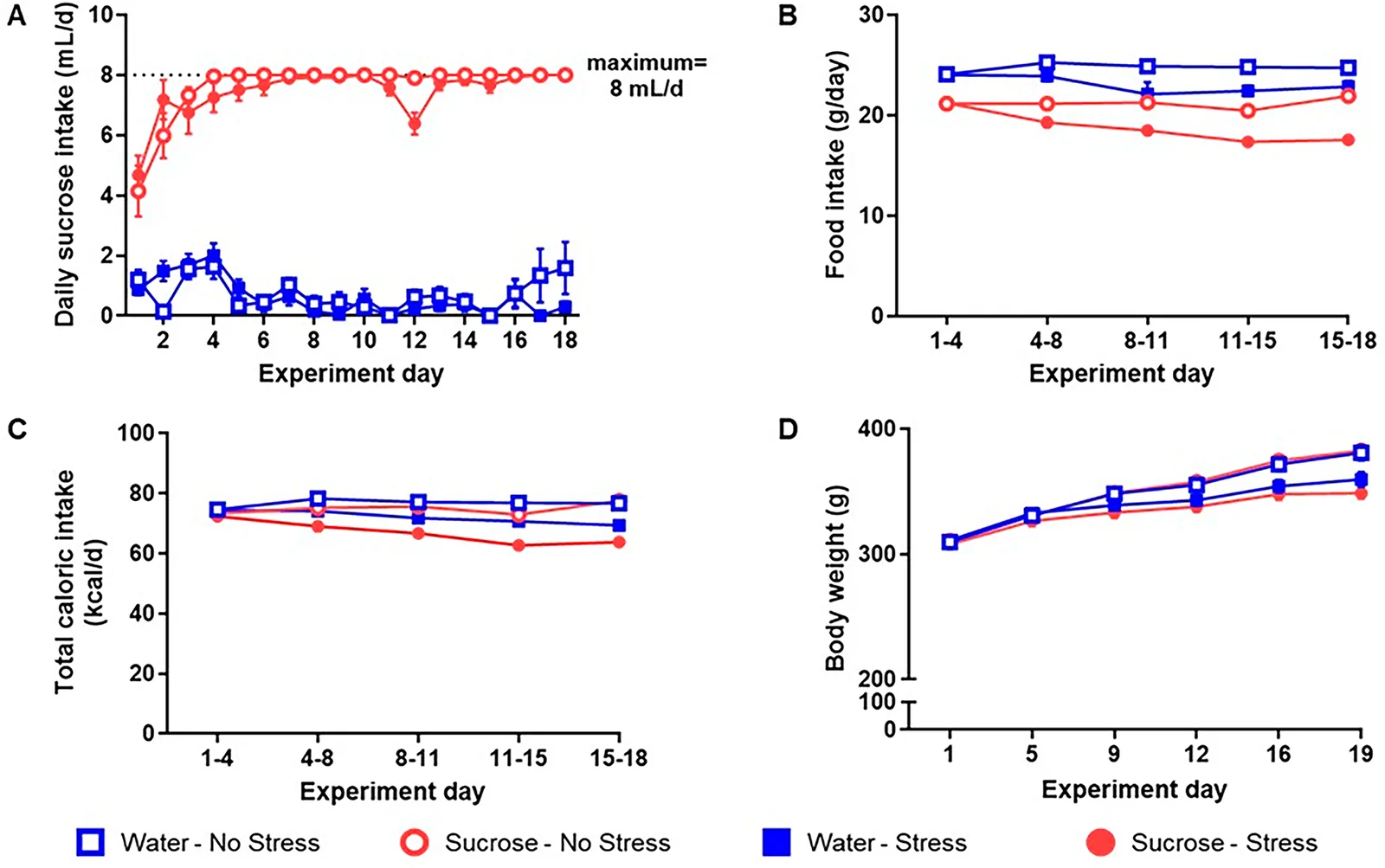
Effects of limited sucrose intake (LSI) and repeated restraint stress on feeding behavior, caloric intake, and body weight. Rats with sucrose access rapidly consumed ~ 8 mL/day (**A**), whereas water controls drank little. Sucrose-fed rats ate less chow (**B**) but maintained total caloric intake (**C**) and body weight (**D**) at control levels. In contrast, stressed rats ate significantly less chow, leading to reduced total caloric intake and body weight compared to unstressed rats. Data were analyzed using 3-way repeated measures ANOVA. Figure reproduced from Nashawi et al., *Physiol Behav*, 2025 (Nashawi et al. 2025)

The data presented in this section have been previously published (Nashawi et al. 2025) and are reproduced here for context and to confirm that the LSI and stress paradigms used in the present study produced their expected effects. Rats with access to 30% sucrose rapidly consumed near-maximal amounts (~ 8 mL/day) regardless of stress, confirming the rewarding properties of sucrose (Fig. 2A). Additionally, rats given sucrose reduced their chow consumption (Fig. 2B) but maintained overall caloric intake (Fig. 2C) and body weight (Fig. 2D) at levels comparable to water controls. In contrast, stressed rats consumed significantly less chow than controls, leading to reduced overall caloric intake and lower body weight compared to unstressed rats. This aligns with prior findings that stress preferentially suppresses consumption of less palatable foods (Pecoraro et al. 2004; Packard et al. 2014; Buesing et al. 2025), and indicates that the repeated restraint paradigm used herein effectively elicited typical stress responses.

**Fig. 3:**
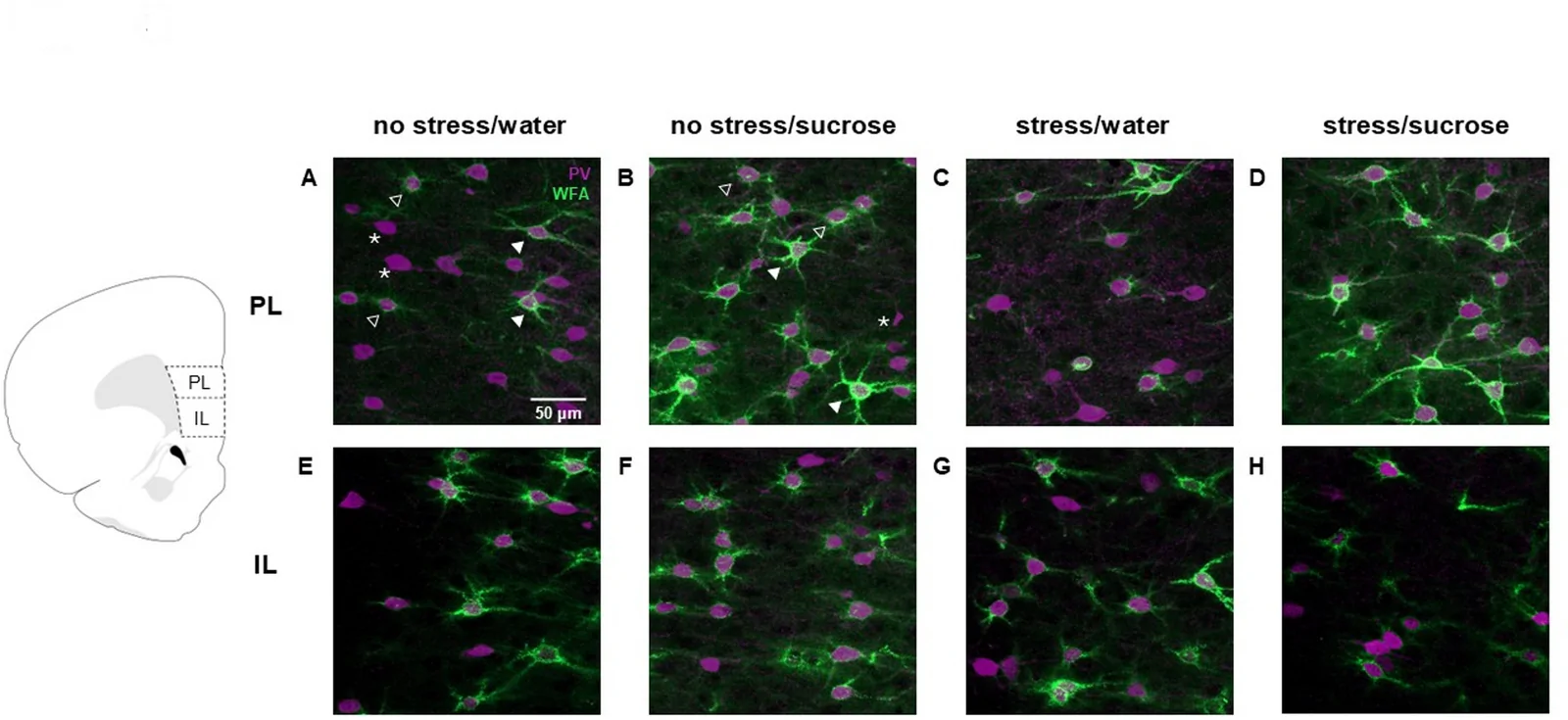
Representative confocal images of PV+ interneurons and perineuronal nets (PNNs) in the prelimbic (PL; **A**-**D**) and infralimbic (IL; **E**-**H**) cortices from rats in each experimental group: no stress/water, no stress/sucrose, stress/water, and stress/sucrose. The images also illustrate the two categories of PNNs analyzed in the present study: full and partial. PNNs were classified as full when WFA labeling completely surrounded the soma and extended onto proximal dendrites, and as partial when one or both of these criteria were not met. Solid white arrowheads indicate PV+ cells surrounded by full PNNs, while open arrowheads mark PV+ cells with partial PNN coverage; both were classified as PV+/WFA+. Asterisks denote PV+ cells without detectable PNN labeling (PV+/WFA−). An inset showing the approximate locations of the PL and IL regions where images were acquired is adapted from the open-access rat brain atlas *Brain Maps 4.0* (Swanson 2018). Scale bar: 50 μm

### Effect of LSI and repeated restraint stress on PV+ interneurons and their PNNs in the prefrontal cortex

**Fig. 4:**
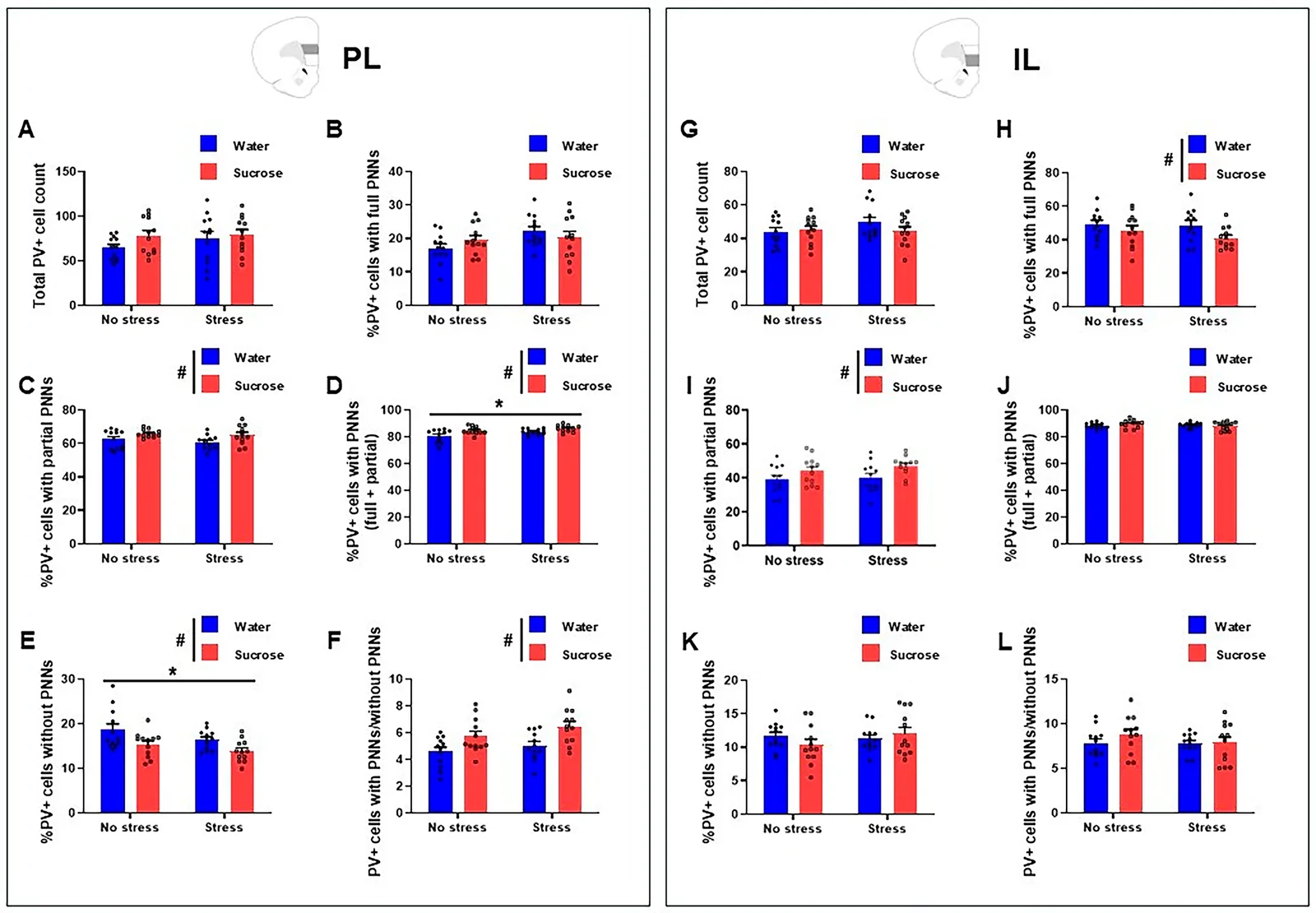
Effects of limited sucrose intake (LSI) and repeated restraint stress on parvalbumin (PV+) cell populations and their perineuronal net (PNN) investment in the prelimbic (PL; **A**-**F**) and infralimbic (IL; **G**-**L**) prefrontal cortices. In the PL, the total number of PV+ cells was unaffected by LSI or restraint stress (**A**), as was the proportion of PV+ cells surrounded by full PNNs (**B**). However, sucrose intake increased the proportion of PV+ cells with partial PNNs (**C**), and both sucrose and stress increased the overall proportion of PV+ cells with any type of PNN (**D**). Both stress exposure and sucrose consumption reduced the proportion of PV+ cells lacking PNNs (**E**); however, only sucrose consumption had a significant effect on the ratio of PV+ cells surrounded by PNN structures (**F**). In the IL, neither LSI nor restraint stress affected the total PV+ cell count (**G**), similar to the PL. Unlike in the PL, however, sucrose consumption significantly reduced the proportion of PV+ cells with full PNNs (**H**) while simultaneously increasing the proportion of PV+ cells with partial PNNs (**I**). This shift in the distribution of PNNs resulted in no net change in the proportion of PV+ cells with (**J**) or without (**K**) PNNs, and therefore the ratio of PV+ cells with to those without PNNs remained stable across all groups (**L**). Data were analyzed using 2-way ANOVAs and are presented as mean ± SEM. * indicates a significant main effect of STRESS; # indicates significant main effect of DRINK. No significant DRINK × STRESS interactions were observed for any parameter. *n* = 12 rats/group

Representative images of PV+ interneurons and their associated PNNs in the PL and IL cortices from each experimental group are shown in Fig. 3A–D (PL) and 3E–H (IL). In the PL, neither sucrose consumption nor stress exposure significantly altered total PV+ cell numbers (Fig. 4A; no main effect of DRINK: F_1,44_ = 2.116, *p* = 0.153; no main effect of STRESS: F_1,44_ = 0.911, *p* = 0.345). This lack of effect may reflect the specific parameters of the stress and reward exposure used in the present study, and importantly, it allowed us to isolate the effects of LSI and stress on PNN modulation without potential confounds related to changes in PV+ cell population size. We next investigated the effects of sucrose and stress exposure on the expression of different PNN forms within this cell population. The proportion of PV+ cells with full PNNs was unaffected by LSI or by stress (Fig. 4B; no main effect of DRINK: F_1,44_ = 0.029, *p* = 0.865 or STRESS: F_1,44_ = 3.728, *p* = 0.06), However, sucrose consumption produced a modest but statistically significant increase in the proportion of PV+ cells with partial PNNs (Fig. 4C; main effect of DRINK: F_1,44_ = 8.53, *p* = 0.006). These changes led to a small increase in the overall proportion of PV+ cells exhibiting any form of PNN (full or partial), with main effects of both STRESS (Fig. 4D; F_1,44_ = 5.815, *p* = 0.02) and DRINK (Fig. 4D; F_1,44_ = 12.24, *p* = 0.001). Sucrose intake also decreased the proportion of PV+ cells lacking PNNs by approximately 17% (Fig. 4E; main effect of DRINK: F_1,44_ = 10.10, *p* = 0.003), with stress producing a smaller but similar reduction of about 11% (Fig. 4E; F_1,44_ = 4.234, *p* = 0.046). This shift resulted in a higher ratio of PV+ cells with PNNs relative to those without, an effect driven primarily by sucrose (Fig. 4F; main effect of DRINK: F_1,44_ = 13.41, *p* = 0.001), with no significant effect of stress (Fig. 4F; F_1,44_ = 2.792, *p* = 0.102). There were no significant DRINK × STRESS interactions across all parameters (*p* > 0.05). These results suggest that LSI increased PV+ cell PNN coverage in the PL, as evidenced by a transition from PNN-lacking to partial PNNs, with stress exerting a minor influence.

Similar to the PL, neither sucrose nor stress influenced total PV+ cell numbers in the IL (Fig. 4G; F_1,44_ = 0.928, *p* = 0.341; F_1,44_ = 0.633, *p* = 0.43, respectively). However, unlike in the PL, sucrose intake significantly reduced the proportion of PV+ cells with full PNNs by approximately 12% (Fig. 4H; main effect of DRINK: F_1,44_ = 5.088, *p* = 0.029). Concurrently, sucrose consumption increased the proportion of PV+ cells with partial PNNs by approximately 15% (Fig. 4I; main effect of DRINK: F_1,44_ = 6.982, *p* = 0.011). The opposing effects of sucrose on full and partial PNNs of PV+ cells in the IL resulted in no net change in the total proportion of PV+ cells with PNNs (combining both full and partial; Fig. 4J), leaving the complementary proportion of PV+ cells devoid of PNNs unchanged as well (Fig. 4K; no main effect of DRINK: F_1,44_ = 0.149, *p* = 0.701). Consequently, the ratio of PV+ cells with PNNs to those without PNNs remained unaffected by sucrose consumption (Fig. 4L; no main effect of DRINK: F_1,44_ = 0.876, *p* = 0.354). Notably, stress had no significant effect on any of the parameters measured in the IL, nor were there any significant DRINK × STRESS interactions (all *p* > 0.05). Collectively, these data show that sucrose intake shifted PNN investment from full to partial forms, indicating reduced PNN structural complexity in the IL.

### Effect of PNN investment on PV+ cell properties and perisomatic puncta apposing PV+ cells in the prefrontal cortex

**Fig. 5:**
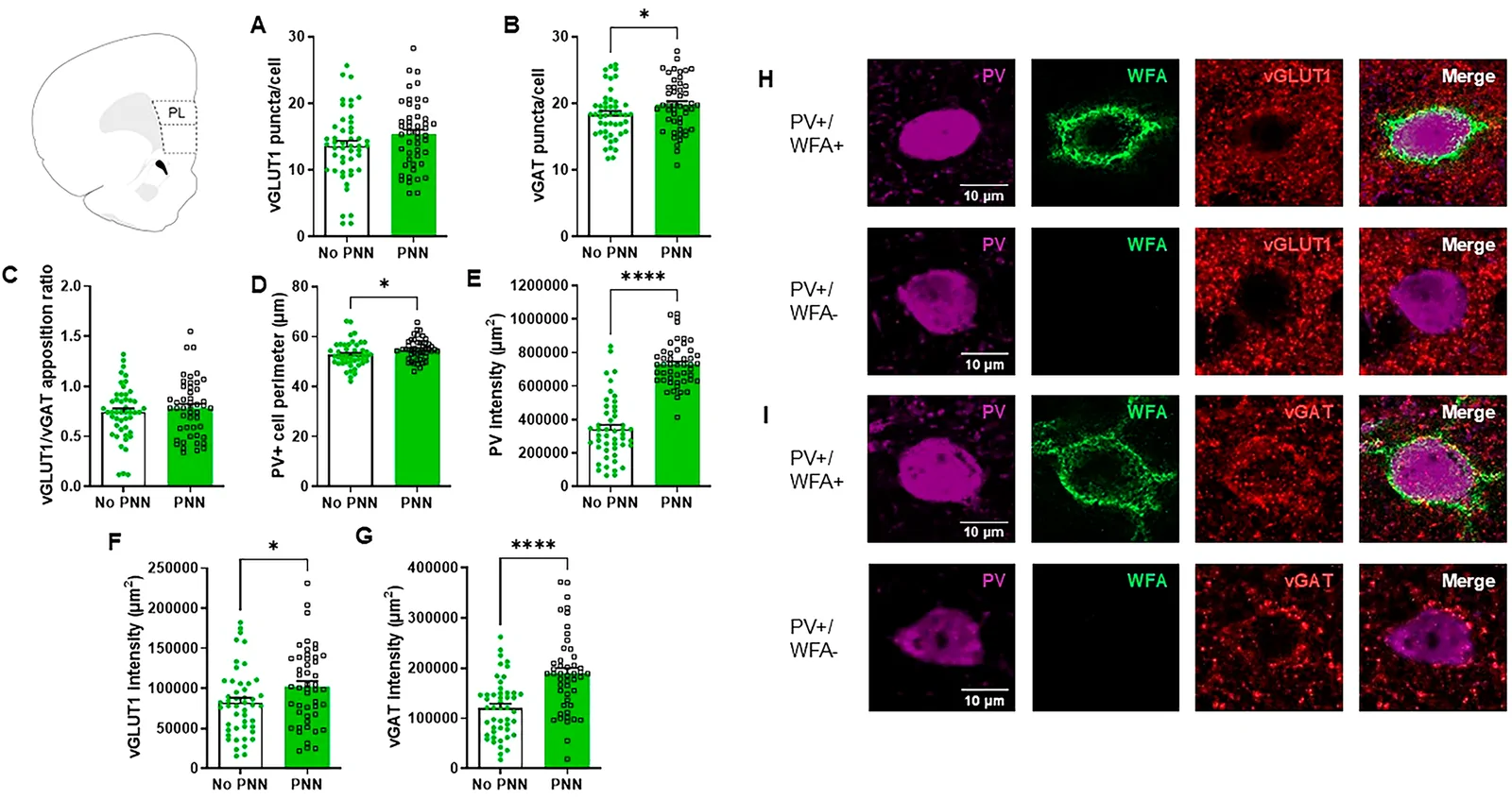
Quantification of perisomatic appositions and cell properties of parvalbumin interneurons (PV+) with and without perineuronal nets (PNNs) in the prelimbic (PL) prefrontal cortex. The number of vGLUT1 + appositions per PV+ cell does not significantly differ between those with and without PNNs (**A**; *p* > 0.05). However, PV+ cells with PNNs receive significantly more vGAT+ puncta per cell compared to those lacking PNNs (**B**; *p < 0.05). Despite this change, the ratio of vGLUT1 + to GAT+ puncta apposing PV+ cells in the PL was similar regardless of PNN status (**C**; *p* > 0.05). PV+ cells with PNNs had larger soma (**D**; *p < 0.05), and showed greater PV intensity (**E** ****p < 0.0001) as well as higher vGLUT1 (**F** *p < 0.05) and vGAT (**G**; *p < 0.001) fluorescence intensity in appositions surrounding the soma. Data represent animal-level means (values from PV+ cells were averaged within each animal; 4 cells per condition per animal) and are presented as mean ± SEM. Statistical analyses were performed using paired t-tests, with the animal as the unit of analysis (*n* = 48 rats, collapsed across experimental conditions). Panels **H** and **I** show representative confocal images of PV+ interneurons (magenta) in the PL, with (top) and without (bottom) PNNs (green), shown with excitatory (vGLUT1+; **H**) or inhibitory (vGAT+; **I**) puncta (red), respectively. Scale bar: 10 μm

Given the critical role of PNNs in regulating neuronal excitability and plasticity, we examined whether their presence was associated with differences in excitatory and/or inhibitory appositions onto PV+ cells in the mPFC, independent of treatment effects. Therefore, data from all treatment groups were combined, and paired t-tests compared PV+ cells with and without PNNs. In the PL, the number of vGLUT1 appositions per cell did not significantly differ between PV+ cells with PNNs and those without (Fig. 5A; *p* = 0.066). However, PV+ cells with PNNs received significantly more vGAT appositions per cell than those without (Fig. 5B; *p* = 0.018), suggesting greater inhibitory input onto PNN-surrounded PV+ neurons. Despite this increase in inhibitory inputs, the ratio of excitatory (vGLUT1+) to inhibitory (vGAT+) appositions onto PV+ cells did not differ based on PNN presence (Fig. 5C; *p* = 0.364). Notably, PV+ cells with PNNs were characterized by larger soma (Fig. 5D; *p* = 0.011), higher PV intensity (Fig. 5E; *p* < 0.0001, paired t-test), and increased vGLUT1 and vGAT staining intensity (Fig. 5F; *p* = 0.012 and Fig. 5G; *p* < 0.0001, respectively). In summary, PNN-associated PV+ cells exhibited larger soma and more intense excitatory and inhibitory perisomatic puncta, without a shift in their relative ratio.

**Fig. 6:**
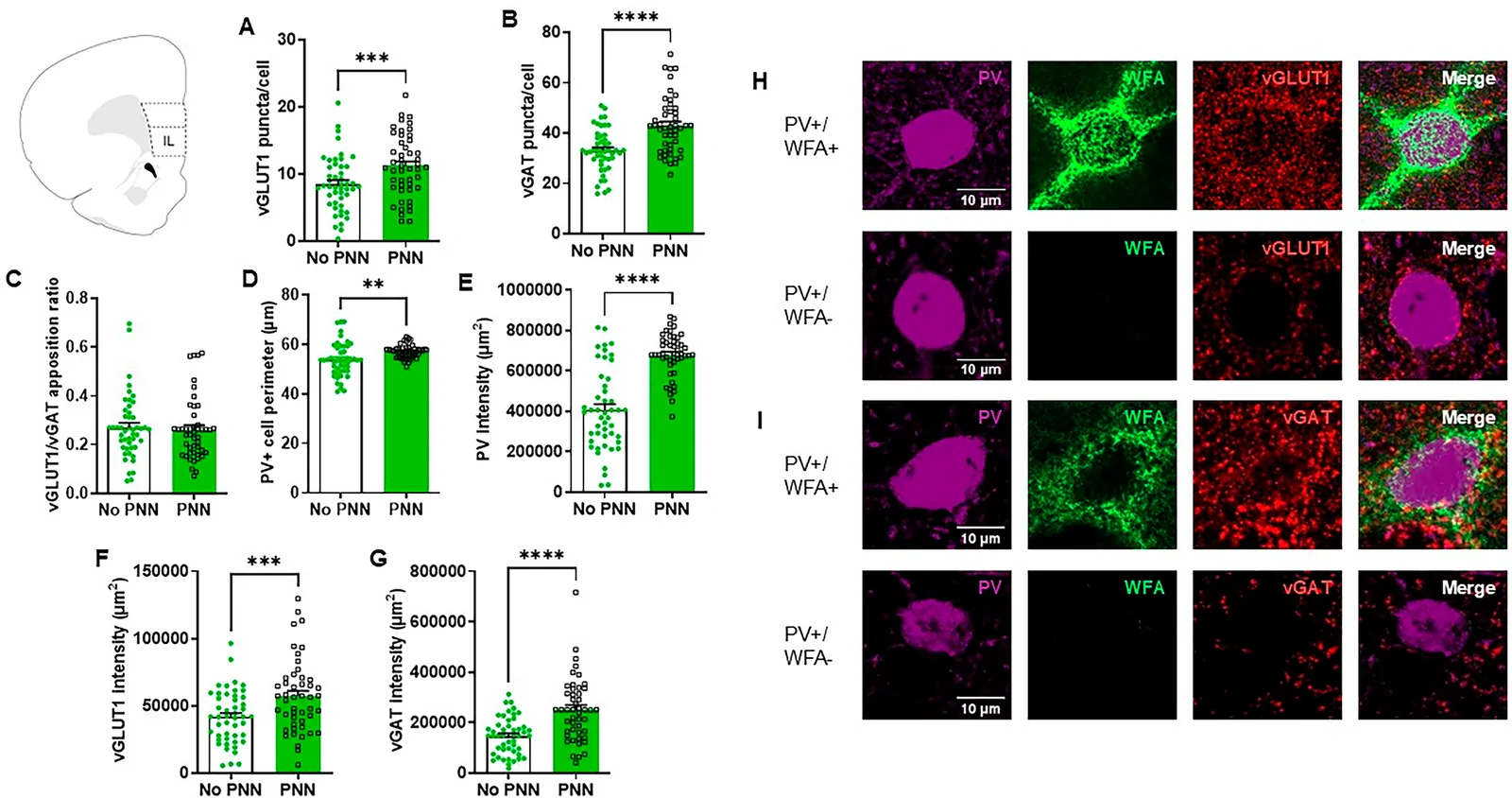
Comparison of perisomatic appositions and cell properties in parvalbumin interneurons (PV+) with and without perineuronal nets (PNNs) in the infralimbic (IL) prefrontal cortex. PV+ cells surrounded by PNNs in the IL receive significantly more vGLUT1+ (**A**; ***p < 0.001) and vGAT+ appositions (**B**; ****p < 0.0001) than their PNN-lacking counterparts. This resulted in an unchanged ratio of excitatory (vGLUT1+) to inhibitory (vGAT+) boutons apposing onto PV+ cells surrounded by PNNs compared to those without PNNs (**C**; *p* > 0.05). Similar to the PL, the cell bodies of PNN-enveloped PV+ cells in the IL are significantly larger than those devoid of PNNs (**D**; **p < 0.01). They also have a greater intensity of labeling for PV (**E**; ****p < 0.0001) and vGLUT1 (**F**; **p < 0.01) and vGAT (**G** ****p < 0.0001) appositions. Data represent animal-level means (values from PV+ cells were averaged within each animal; 4 cells per condition per animal) and are presented as mean ± SEM. Statistical analyses were performed using paired t-tests, with the animal as the unit of analysis (*n* = 48 rats, collapsed across experimental conditions). Panels **H** and **I** show representative confocal images of PV+ interneurons (magenta) in the IL, with (top) and without (bottom) PNNs (green), shown with excitatory (vGLUT1+; **H**) or inhibitory (vGAT+; **I**) puncta (red), respectively. Scale bar: 10 μm

In the IL, PV+ cells with PNNs exhibited significantly more appositions compared to those without PNNs, with increases in both vGLUT1+ (Fig. 6A; *p* = 0.001) and vGAT+ (Fig. 6B; *p* < 0.0001) puncta per cell. The simultaneous increase in both excitatory and inhibitory inputs onto PV+ cells with PNNs rendered the vGLUT1/vGAT ratio in PV+ cells with PNNs unchanged compared to those without PNNs (Fig. 6C; *p* = 0.997). Consistent with our observations in the PL, cells with PNNs in the IL were larger (Fig. 6D; *p* = 0.017, paired t-test), demonstrated higher PV intensity (Fig. 6E; *p* < 0.0001, paired t-test), and exhibited increased vGLUT1 and vGAT staining intensity (Fig. 6F; *p* < 0.001 and Fig. 6G; *p* < 0.0001, respectively). The effects of PNN investment on PV+ cell properties and excitatory and inhibitory appositions in the PL and IL are summarized in Table 1. For comparison, corresponding BLA data from our previous report (Nashawi et al. 2025) are also included. vGLUT1 + and vGAT+ apposition intensities in the BLA are additional analyses that were not previously published and are shown here for the first time in Table 1 and Supplementary Material 1. 

**Table 1:** Effect of perineuronal net (PNN) presence on parvalbumin (PV+) interneuron properties and putative synaptic connectivity in the prelimbic (PL) and infralimbic (IL) regions of the medial prefrontal cortex (mPFC), compared to the basolateral amygdala (BLA; from (Nashawi et al. 2025)

Measure	PL-mPFC	IL-mPFC	BLA
*1) PV+ cell properties*			
PV+ cell perimeter	↑	↑	↑
PV intensity	↑	↑	↑
*2) vGLUT1 + appositions*			
Apposition number	=	↑	↑
Intensity	↑	↑	↑ ^a^
*3) vGAT+ appositions*			
Apposition number	↑	↑	↓
Intensity	↑	↑	↑ ^a^
*4) vGLUT1/vGAT ratio*	=	=	↑

^a^ previously unpublished data that is now shown in Supplementary Material 1

### Effect of LSI and repeated restraint stress on the perisomatic puncta apposing PV+ cells in the prefrontal cortex

**Fig. 7:**
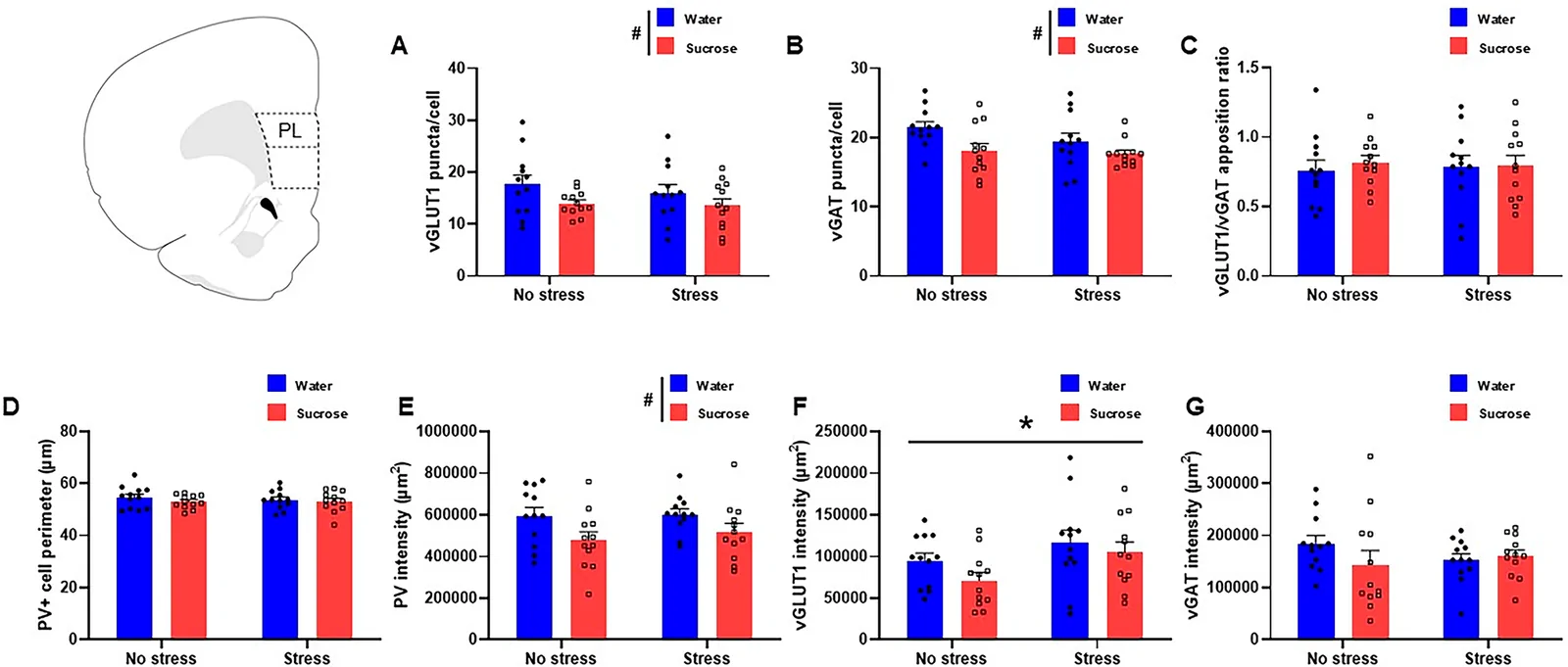
Effects of LSI and repeated restraint stress on perisomatic appositions and cellular properties of (PV+) parvalbumin interneurons in the prelimbic (PL) prefrontal cortex. Sucrose consumption significantly reduced the number of vGLUT1 + puncta apposing PV+ cells (**A**). Similarly, sucrose decreased vGAT+ puncta apposing PV+ cells (**B**). Despite these reductions in both excitatory and inhibitory inputs, the vGLUT1/vGAT ratio remained unchanged across treatment conditions (**C**). PV+ cell size, as measured by PV+ cell perimeter, was not significantly affected by either intervention (**D**). Unexpectedly, sucrose intake significantly decreased PV protein expression in these interneurons (**E**). Stress exposure, but not sucrose consumption, increased perisomatic puncta labeling intensities for excitatory markers (**F**); however, inhibitory markers were unaffected by either treatment (**G**). Data were analyzed using two-way ANOVAs and are presented as mean ± SEM. * indicates a significant main effect of STRESS; # indicates significant main effect of DRINK. No significant DRINK × STRESS interactions were observed for any parameter. *n* = 12 rats/group

To further dissect the effects of sucrose and stress exposure on PV+ cell properties and putative connectivity in the mPFC, we conducted 2-way ANOVA analyses with DRINK and STRESS as between subject factors. When examining treatment effects in the PL, we found that sucrose consumption significantly reduced both excitatory and inhibitory appositions onto PV+ cells (Fig. 7A and B, respectively), decreasing vGLUT1+ (main effect of DRINK: F_1,44_ = 4.672, *p* = 0.036) and vGAT+ puncta per cell (main effect of DRINK: F_1,44_ = 7.833, *p* = 0.008). However, the ratio of excitatory vs. inhibitory puncta apposing PV+ cells remained unchanged by sucrose (Fig. 7C; no main effect of STRESS: F_1,44_ = 0.0003 or DRINK: F_1,44_ = 0.199). While PV+ cell perimeter was not significantly altered by stress or sucrose intake (Fig. 7D; no main effect of STRESS: F_1,44_ = 0.112 or DRINK: F_1,44_ = 1.126), LSI significantly decreased PV protein expression in these interneurons, measured by PV fluorescence intensity (Fig. 7E; main effect of DRINK: F_1,44_ = 7.368, *p* = 0.009). In contrast, neither excitatory nor inhibitory terminal staining intensities were affected by LSI (Fig. 7F and G). Additionally, stress exposure increased vGLUT1 intensity (Fig. 7F; main effect of STRESS: F_1, 44_ = 5.281, *p* = 0.026). In summary, sucrose diminished vGLUT + and vGAT+ presynaptic terminals apposing onto PV+ cells in the PL and reduced PV protein expression, while stress enhanced excitatory terminal staining independent of apposition number.

**Fig. 8:**
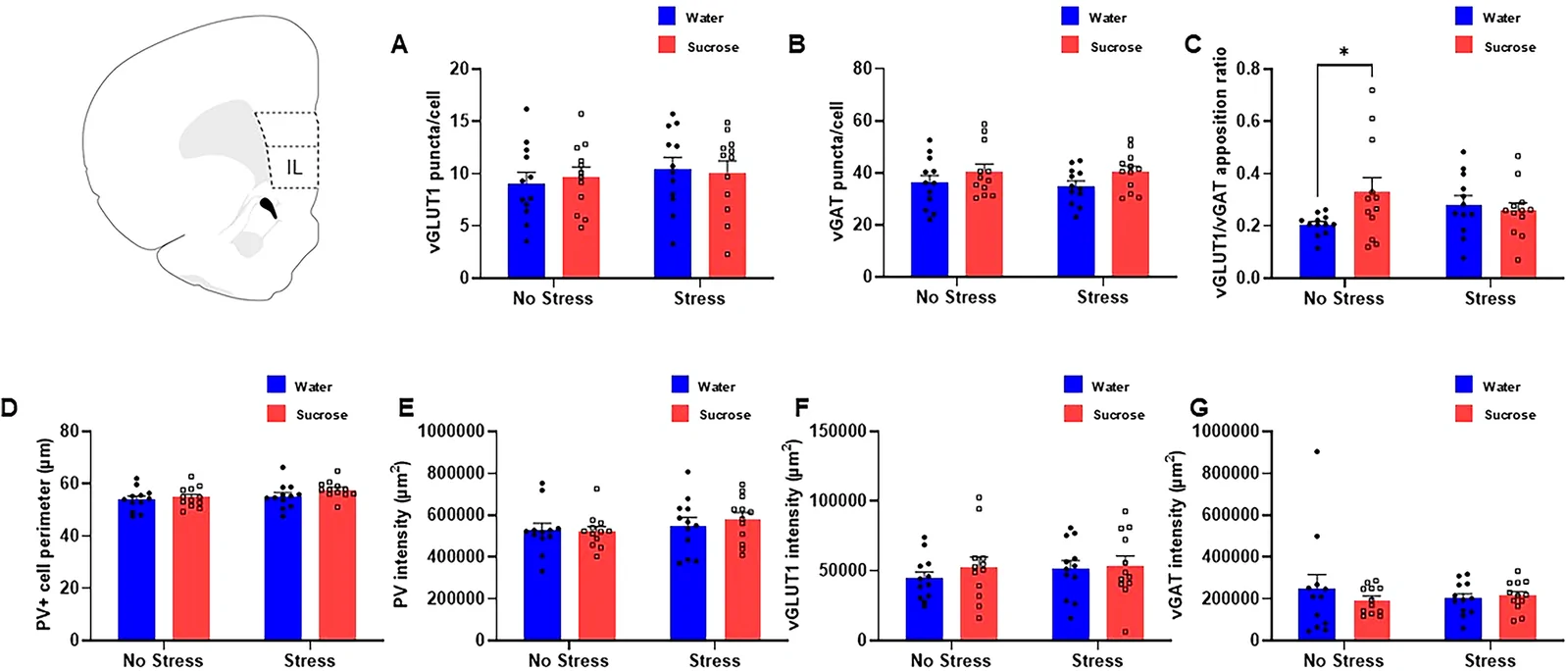
Analysis of perisomatic appositions and cellular properties of parvalbumin (PV+) interneurons in the infralimbic (IL) prefrontal cortex following sucrose consumption and stress exposure. Neither LSI or repeated restraint stress affected the number of vGLUT1+ (**A**) or vGAT+ (**B**) puncta apposed to PV+ cells. The vGLUT1/vGAT apposition ratio was modulated by a DRINK × STRESS interaction, with sucrose effects limited to the No Stress group (**C**). Cell morphology parameters, including PV+ cell perimeter (**D**) and the intensity of PV protein labeling (**E**), were not significantly affected by stress exposure or sucrose consumption. Staining intensities for both excitatory (**F**) and inhibitory (**G**) perisomatic puncta remained stable across all groups. Data were analyzed using two-way ANOVAs and are presented as mean ± SEM. No significant main effects of DRINK or STRESS were observed for any parameter. *n* = 12 rats/group

Interestingly, in contrast to the changes observed in the PL, two-way ANOVAs revealed no significant main effects of either stress exposure or sucrose intake on perisomatic appositions onto PV+ cells the IL (Fig. 8; all *p* > 0.05). However, a significant DRINK × STRESS interaction was observed for the vGLUT1/vGAT apposition ratio (Fig. 8C; F_1, 44_ = 4.147, *p* = 0.048), with post-hoc tests revealing that sucrose significantly increased the vGLUT1/vGAT ratio compared to water in the No Stress group, but not in the stressed group. Collectively, these results suggest mPFC subregion-specific effects of these factors on putative synaptic inputs. The effects of LSI on PV+ cell properties and appositions in the PL and IL are summarized in Table 2, providing an overview of how these measures vary as a function of sucrose exposure and complementing the PNN-based comparisons shown in Table 1. BLA data is included for comparison. While vGLUT1 + and vGAT+ apposition counts and their ratio were reported in [70], all other BLA measures are newly analyzed and appear in Table 2 and Supplementary Material 2.

**Table 2:** Effect of prior sucrose exposure on parvalbumin (PV+) cell properties in prelimbic (PL) and infralimbic (IL) medial prefrontal cortex (mPFC), compared to the basolateral amygdala (BLA; from (Nashawi et al. 2025)

Measure	PL-mPFC	IL-mPFC	BLA
*1) PV+ cell properties*			
PV+ cell count	=	=	=
PV+ cell perimeter	=	=	= ^a^
PV intensity	↓	=	= ^a^
*2) PNN coverage*	↑ (none → partial)	↓ (full → partial)	↑ (none → partial)
*3) vGLUT1 + appositions*			
Apposition number	↓	=	=
Intensity	=	=	= ^a^
*3) vGAT+ appositions*			
Apposition number	↓	=	=
Intensity	=	=	= ^a^
*4) vGLUT1/vGAT ratio*	=	↑ (only in No Stress group)	↑

^a^ previously unpublished data that is now shown in Supplementary Material 2

**Fig. 9:**
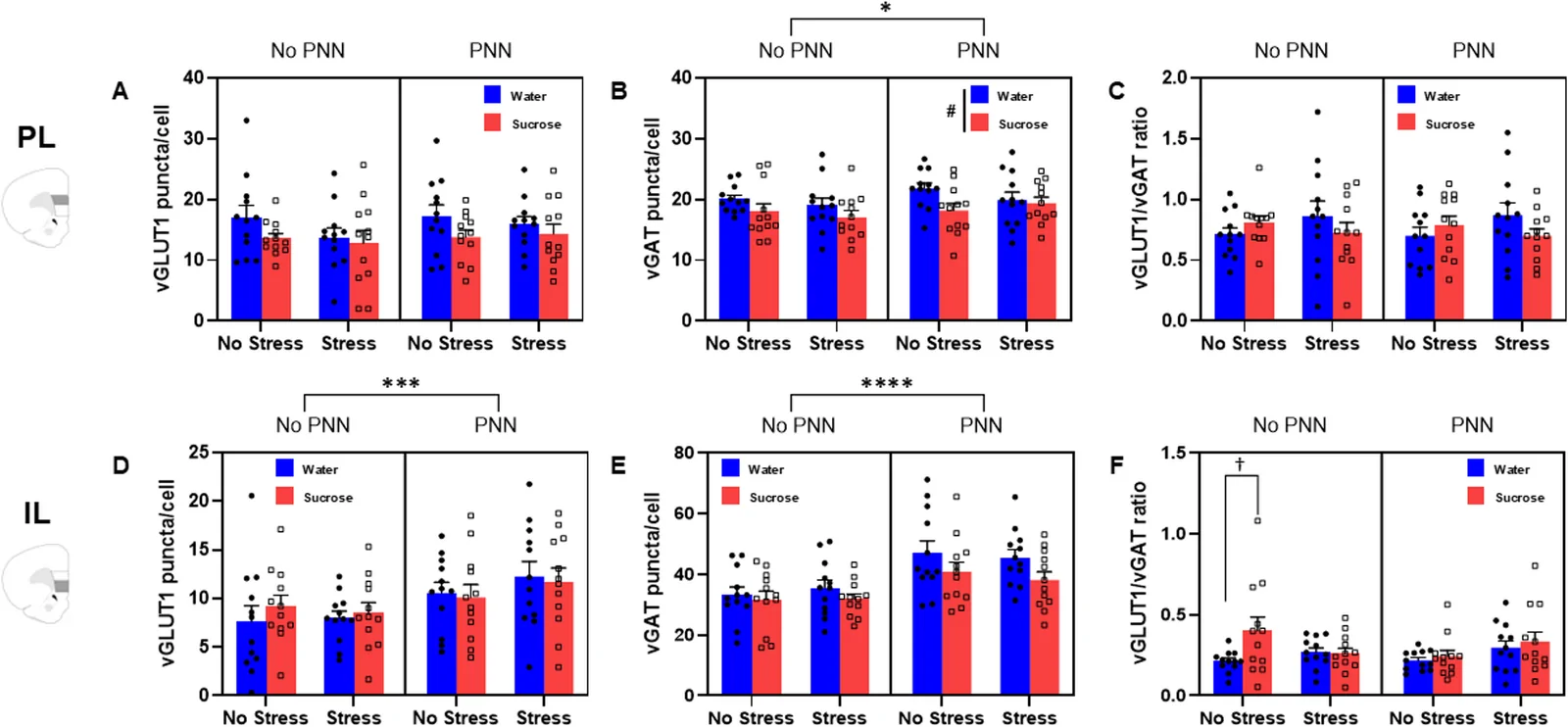
Three-way analysis of perisomatic appositions onto parvalbumin (PV+) interneurons in the prelimbic (PL) and infralimbic (IL) prefrontal cortex as a function of perineuronal net (PNN) presence, sucrose consumption, and stress exposure. In the PL, sucrose consumption did not alter the number of vGLUT1+ puncta apposing PV+ cells regardless of stress exposure or PNN presence (A). Sucrose consumption reduced vGAT+ apposition number, while PNN presence increased it (B). The vGLUT1/vGAT ratio was not modulated by any factor (C). In the IL, PNN presence increased both vGLUT1+ (D) and vGAT+ (E) apposition numbers, with no significant effects of sucrose or stress on either measure. The IL vGLUT1/vGAT ratio (F) was modulated by significant STRESS × PNN PRESENCE and DRINK × STRESS × PNN PRESENCE interactions, such that sucrose increased the ratio in PNN-negative PV+ cells in unstressed rats; this effect was absent after repeated restraint stress and was not observed in PNN-bearing cells. Data were analyzed using three-way ANOVAs (DRINK × STRESS × PNN PRESENCE) with PNN PRESENCE included as a repeated-measures factor and are presented as mean ± SEM. # indicates a significant main effect of DRINK; * indicates a significant main effect of PNN presence; † indicates a significant pairwise comparison (#, *, or † p < 0.05, **p < 0.01, ***p < 0.001, ****p < 0.0001). n = 12 rats/group

To determine whether the effects of sucrose intake and stress on excitatory and inhibitory perisomatic appositions onto PV+ neurons were secondary to changes in PNN ensheathment, three-way ANOVAs (DRINK × STRESS × PNN PRESENCE) were conducted separately for the PL and IL regions of the mPFC, with PNN PRESENCE included as a repeated-measures factor. In the PL, PNN presence significantly increased vGAT apposition count (F_1,44_ = 4.984, *p* = 0.031), while sucrose consumption had the opposite effect (F_1,44_ = 4.62, *p* = 0.037). In the IL, PNN presence significantly increased both vGLUT and vGAT apposition numbers (F_1,44_ = 14.5, *p* < 0.001; and F_1,44_ = 48.43, *p* < 0.0001, respectively). There was also a significant STRESS × PNN PRESENCE interaction on the vGLUT1/vGAT ratio of PV+ interneurons (F_1,44_ = 6.627, *p* = 0.014), as well as a significant DRINK × STRESS × PNN PRESENCE interaction (F_1,44_ = 4.492, *p* = 0.04). These interactions indicate that the impact of sucrose on the vGLUT1/vGAT ratio depended on both stress history and the presence of PNNs. While sucrose increased the ratio in PNN-negative PV+ cells in unstressed rats, this effect was absent after repeated restraint stress. This effect was seen only in PNN-negative cells, suggesting a relative stability of the vGLUT1/vGAT ratio in PNN-associated cells. There were no other significant main or interactive effects of DRINK or STRESS in either region (all *p* > 0.05). The three-way ANOVA analysis outcomes are summarized in Fig. 9; Table 3. Table 3 also includes previously published BLA data (Nashawi et al. 2025) for reference.

**Table 3:** Summary of 3-way analysis of variance (ANOVA) comparing the effects of perineuronal net (PNN) presence, limited sucrose intake (LSI), and repeated restraint stress on excitatory (vGLUT1+) and inhibitory (vGAT+) appositions onto parvalbumin (PV+) interneurons across the prelimbic (PL) and infralimbic (IL) medial prefrontal cortices (mPFC) and the basolateral amygdala (BLA; from (Nashawi et al. 2025)

Measure	DRINK (D)	STRESS (S)	PNN Presence (*P*)	Interactions
*PL-mPFC*				
vGLUT1 apposition number	ns	ns	ns	ns
vGAT apposition number	↓*	ns	↑*	ns
vGLUT1/vGAT ratio	ns	ns	ns	ns
*IL-mPFC*				
vGLUT1 apposition number	ns	ns	↑***	ns
vGAT apposition number	ns	ns	↑****	ns
vGLUT1/vGAT ratio	ns	ns	ns	S × P * D × S × P *
*BLA*				
vGLUT1 apposition number	ns	ns	↑**	ns
vGAT apposition number	ns	ns	↓***	ns
vGLUT1/vGAT ratio	ns	ns	↑***	ns

ns = not significant; * *p* < 0.05; ** *p* < 0.01; *** *p* < 0.001, **** *p* < 0.0001

## Discussion

To gain insight into how natural rewards promote stress resilience, we examined the structural plasticity of PV+ interneurons in the mPFC following LSI and repeated restraint stress, focusing on PNN organization and perisomatic puncta. In both subregions, the principal effect of LSI is best understood as a proportional redistribution of PV+ interneurons among PNN states, rather than a large-scale change in the absolute number of cells bearing PNNs. In the PL-mPFC, LSI increased the proportion of PNN-ensheathed PV+ cells, primarily by shifting cells from PNN-lacking to a partially ensheathed state. In contrast, in the IL-mPFC, LSI decreased fully ensheathed PV+ cells and increased those with partial PNNs, without affecting overall PNN prevalence. These differential effects of LSI on PNN form are particularly notable given that PNNs are increasingly recognized to be structurally heterogeneous (Pollock et al. 2014; Sigal et al. 2019), and that changes in PNN completeness may shape the balance between stability and plasticity in PV+ interneuron circuits (Sigal et al. 2019). Fully condensed PNNs constrain synaptic remodeling and promote network stability, while partial PNNs are more permissive of activity-dependent reorganization while maintaining structural support (Sigal et al. 2019). This functional distinction is supported by experimental evidence that PNN cleavage reopens cortical plasticity in adult animals (Pizzorusso et al. 2002) and by recent work in the human prefrontal cortex demonstrating that PNN morphological diversity reflects microcircuit specialization in the human prefrontal cortex (Banovac et al. 2025). Given that the PL and IL subregions contribute differently to stress regulation (Herman et al. 2005b; Radley et al. 2006), with the PL exerting inhibitory control over neuroendocrine stress responses and the IL modulating autonomic and long-term adaptive responses to chronic stress (Radley et al. 2006), the LSI-induced subregion-specific changes may have distinct functional outcomes. Increased PNN coverage in the PL may stabilize its inhibitory influence to prevent excessive stress responses, whereas the shift toward partial PNNs in the IL may enhance circuit flexibility supporting adaptive stress recovery and autonomic regulation.

We used repeated restraint stress as our chronic stress model because it is a homotypic stressor, allowing direct comparison with the homotypic reward of LSI. This paradigm is widely used in stress research and it reliably activates HPA axis, autonomic, and behavioral stress responses (Martí et al. 1994; Harris et al. 1998; Chiba et al. 2012; Sikora et al. 2016; Wang et al. 2017). In our study, stressed rats exhibited sustained reductions in chow consumption, total caloric intake, and body weight gain, consistent with established effects of chronic stress (Gamallo et al. 1986; Alario et al. 1987; Martí et al. 1994; Meerlo et al. 1996; Harris et al. 1998; Torres and Nowson 2007; Ulrich-Lai et al. 2007; Chiba et al. 2012) and supporting the effectiveness of our protocol. Despite these systemic indicators of chronic stress, repeated restraint had minimal impact on PNNs and PV+ cells in the mPFC, an intriguing finding given prior reports of stress-induced PNN remodeling in multiple brain regions (Spijker et al. 2020; Perlman et al. 2021; Laham and Gould 2022; Morphett et al. 2024). This suggests that repeated restraint engages neuronal pathways that exert limited influence on the PNN and PV+ cell properties examined in the mPFC. Habituation to restraint (Girotti et al. 2006; Grissom and Bhatnagar 2009; Shoji and Mizoguchi 2010; Babb et al. 2014) may have also contributed, though this is unlikely given the persistent metabolic and behavioral deficits throughout the 14-day exposure (however, it cannot be excluded for other, unmeasured endpoints) (Marin et al. 2007). Our findings add to accumulating evidence that the effect of stress on PNNs is highly paradigm-dependent, varying with stressor type, duration, and intensity, as well as with the age and sex of the animals and timing of assessment (Spijker et al. 2020; Perlman et al. 2021; Laham and Gould 2022; Morphett et al. 2024). Studies showing pronounced PNN alterations typically involve juvenile or early-life stress, when the brain exhibits greater plasticity. Our data in adult rats align with reports that repeated restraint does not alter PV+ PNNs number in the adult mPFC (Pesarico et al. 2019).

PNN-bearing PV+ interneurons in the mPFC constitute a morphologically distinct subpopulation. In line with previous reports (Yamada et al. 2015a, b; Carceller et al. 2020; Smail et al. 2023; Nashawi et al. 2025), we found that these cells were larger and exhibited higher PV expression than their PNN-negative counterparts across mPFC subregions, features associated with enhanced calcium buffering and stronger inhibitory output (Dityatev et al. 2007; Slaker et al. 2018). Because PV also regulates synaptic timing and short-term plasticity at fast-spiking interneuron synapses (Orduz et al. 2013), elevated PV levels in this subpopulation likely enhance the temporal precision and reliability of inhibition, consistent with a high-capacity, stable inhibitory phenotype. This was further evidenced by more intensely labelled vGLUT + and vGAT+ appositions, indicating a more mature and synaptically integrated state. Furthermore, PNNs may help preserve PV+ interneuron inhibitory function under stressful conditions by protecting them from oxidative damage (Liu et al. 1996; Morawski et al. 2004; Atif et al. 2008; Suttkus et al. 2012; Cabungcal et al. 2013a, b; Novaes et al. 2018), to which this cell type is particularly vulnerable (Cabungcal et al. 2013a, b). Multiple studies have demonstrated that enhanced PV+ interneuron-mediated inhibition is linked to stress resilience in preclinical models (Holland et al. 2014; Perova et al. 2015; Page and Coutellier 2018; Li et al. 2022; Arime et al. 2024; Santos-Silva et al. 2024), whereas deficient PV+ interneuron activity leads to network destabilization and stress susceptibility (Wang et al. 2014; Li et al. 2022; Arime et al. 2024; Santos-Silva et al. 2024). Therefore, PNN ensheathment defines a stable, high-performing PV+ interneuron subset that may contribute to maintaining prefrontal network balance under stress.

Given the PNN-associated cellular characteristics described above, we expected that the LSI-induced PNN remodeling would alter PV+ cell features accordingly. Instead, we observed a complex, subregion-specific dissociation. In the PL, LSI raised PNN prevalence but did not increase PV+ cell size, intensity, or vGAT+ appositions at the population level, suggesting that the PNN increase was of insufficient magnitude to drive these effects. Furthermore, LSI appeared to exert direct effects on PL PV+ neurons that opposed, and possibly outweighed, PNN-mediated influences, reducing PV intensity and perisomatic appositions. In the IL, LSI reduced PNN complexity but had no effect on PV+ cell properties. These findings suggest that natural reward exerts two distinct and subregion-specific effects in the mPFC: one that remodels PNN structure and another that directly modulates PV+ interneuron properties. Interestingly, LSI increased the excitatory/inhibitory (E/I) apposition ratio in the IL, but only on PNN-negative PV+ cells in stress-naïve rats, indicating that LSI-induced plasticity in the IL is gated by both PNN status and stress history. Our observations add to evidence that natural reward effects on PNNs vary across brain regions, can depend on prior experience, and that PNN remodeling can occur independently of other PV+ cell properties (Brown and Sorg 2022). For example, environmental enrichment bidirectionally modulates PL PNN intensity based on sucrose history (using a paradigm of sucrose self-administration) while selectively increasing IL PNNs only after sucrose exposure (Slaker et al. 2016). Similarly, high-fat, high-sugar diets increase PNN-bearing PV+ cells despite reducing total PV+ interneuron numbers (Reichelt et al. 2019a). Collectively, these findings demonstrate that natural rewards can engage multiple independent mechanisms to remodel prefrontal PV+ interneuron circuits in a region- and experience-dependent manner.

**Fig. 10:**
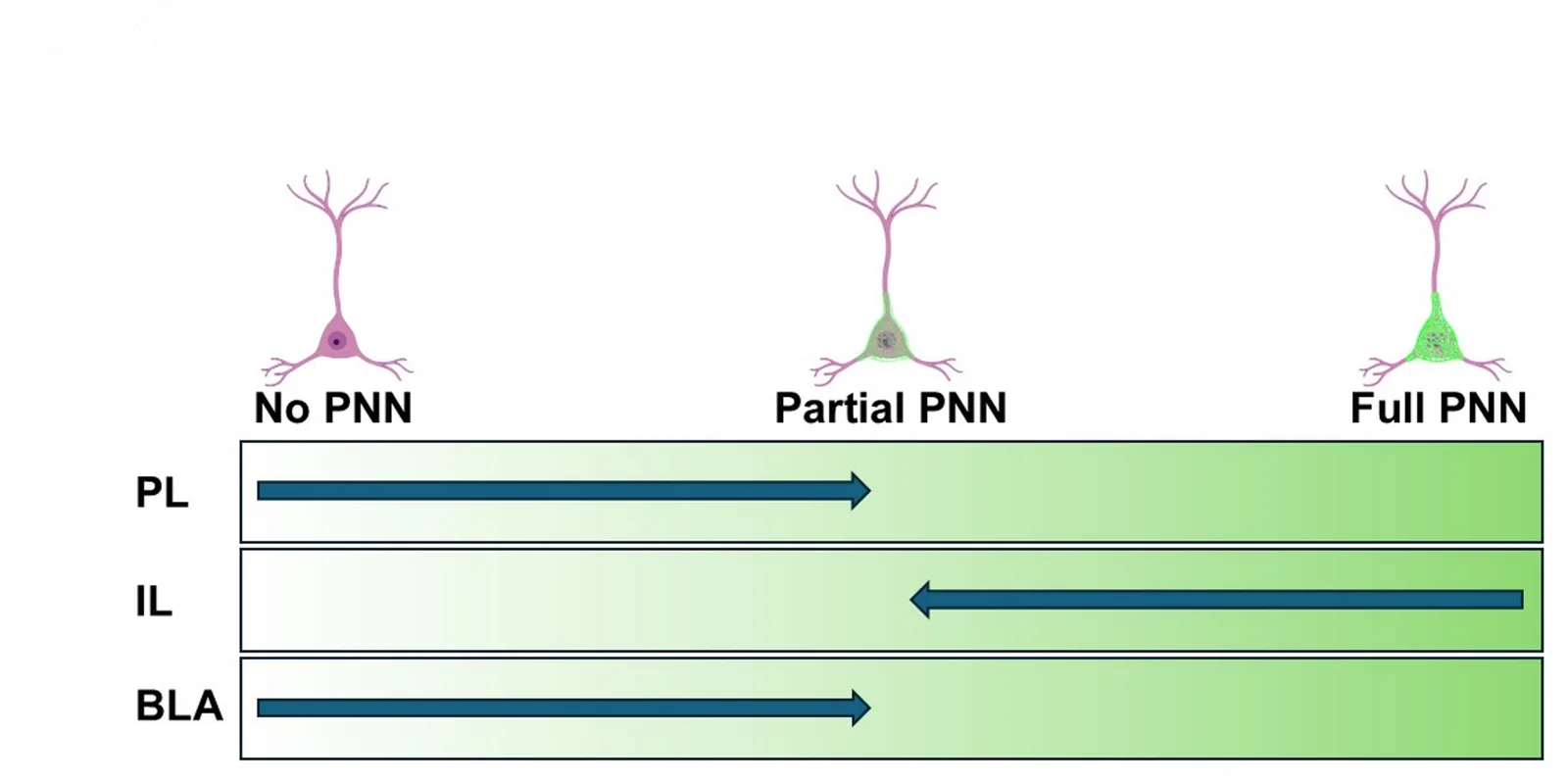
Summary of subregion-specific effects of limited sucrose intake (LSI) on perineuronal nets (PNNs) of parvalbumin (PV+) interneurons in the medial prefrontal cortex (mPFC) and compared to prior findings in the basolateral amygdala (BLA). In the prelimbic (PL) cortex, LSI increased PNN investment on PV+ cells, reflected by a shift from PNN-free to PNN-associated cells. In the infralimbic (IL) cortex, LSI promoted PNN remodeling, shifting from fully formed to more diffuse PNNs, without changing the overall proportion of PV+ cells with PNNs. For comparison, we previously found that LSI increased the proportion of PV+ cells with partial PNNs in the BLA while reducing the proportion of PNN-free PV+ cells (Nashawi et al. 2025), a pattern resembling that reported here for the PL. These region-specific patterns of PNN reorganization suggest that natural reward modulates inhibitory microcircuits in distinct ways across stress- and reward-related brain regions

Our current findings extend our prior work in the BLA (Nashawi et al. 2025), revealing parallels and distinctions between these stress-regulatory structures. Similar to the PL, LSI increased the proportion of PNN-bearing PV+ interneurons in the BLA, primarily by increasing partial PNN coverage. However, unlike the PL, LSI in the BLA raised the vGLUT1/vGAT apposition ratio on PV+ cells, an effect driven by PNN presence rather than a direct effect of LSI. The dissociation between PNN remodeling and PV+ cell morphology seen in the mPFC extended to the BLA, where LSI altered PNN structure but not PV+ cell size or intensity. These findings imply that natural rewards recruit a common form of PNN-mediated plasticity across the BLA and mPFC, potentially stabilizing PV+ interneuron function. However, the region-specific modulation of E/I apposition balance indicates that the consequences of PNN remodeling may differ based on each region’s role in the stress response regulation. In the BLA, the elevated E/I apposition ratio is consistent with a potential for enhanced feedforward inhibition that dampens stress-excitatory output, whereas in the mPFC, maintaining E/I stability may enhance network synchrony and top-down control of stress responses. A further regional distinction emerges in how stress and reward shape these inhibitory circuits. In the BLA, LSI-driven changes in excitatory and inhibitory appositions appear to be mediated entirely by alterations in PNN investment, with stress and natural reward exerting no detectable influence on synaptic apposition prevalence beyond their effects on PNN coverage (Nashawi et al. 2025). In the IL, by contrast, LSI engages additional mechanisms beyond PNN remodeling alone (as discussed above), pointing to a region-specific divergence in how natural reward integrates with stress signaling. These distinctions underscore complementary but distinct mechanisms by which natural rewards shape inhibitory circuitry to promote stress resilience. The region-specific patterns of PNN remodeling induced by LSI in the mPFC and BLA are illustrated in Fig. 10.

While our study identifies a potential PNN-mediated mechanism for natural reward-induced stress resilience, several key questions remain. First, although evidence links PNN integrity and PV+ interneuron function to reduced stress-related behaviors (Wang et al. 2014; Li et al. 2022; Arime et al. 2024; Santos-Silva et al. 2024), this was not examined in the current study. Causal experiments using region- and cell-specific PNN manipulations, such as genetic approaches, are needed to directly test the role of prefrontal PV+ interneurons and their PNNs in natural reward-driven stress buffering. Second, the molecular and cellular mechanisms by which LSI causes prefrontal PNN remodeling remain unclear. PNN integrity is regulated by several ECM–modifying enzymes, such as metalloproteinases and hyaluronidases, produced by both neurons and glial cells (Testa et al. 2019; Bosiacki et al. 2019; Tewari et al. 2022; Soles et al. 2023; Santos-Silva et al. 2024). Identifying the specific pathways and cell types mediating reward-induced PNN plasticity will be critical for understanding how natural rewards engage ECM signaling to modulate inhibitory microcircuits in the mPFC. Third, our study examined only one form of natural reward (sucrose intake) and one stress paradigm (repeated restraint). Whether the effects observed here generalize to other rewarding stimuli or other stressors remains to be determined. Finally, this study was conducted exclusively in male rats, precluding assessment of potential sex differences in reward-induced PNN remodeling. We focused on male rats as first step towards understanding PNN regulation in the mPFC as this allowed us to use tissue from a prior study (Nashawi et al. 2025), thereby minimizing animal usage. Now that LSI-dependent effects have been identified in males, future work will focus on expanding findings to female rats, in which LSI effects on PNN regulation will likely be more complex. PNNs are dynamically regulated across the estrous cycle and by ovarian hormones in multiple brain regions (Uriarte et al. 2020; Zhang et al. 2021; Laham et al. 2022; Hernández-Vivanco et al. 2025; Nguyen et al. 2025). Moreover, the stress-buffering effects of LSI in female rats varies across the estrous cycle (Egan et al. 2018, 2019), suggesting that PNN remodeling could contribute to estrous-cycle-dependent effects of natural reward on stress responses.

In summary, our findings identify PNN-associated plasticity of PV+ interneurons as a novel mechanism through which natural rewards shape prefrontal circuits involved in stress regulation. By doing so, this work extends prevailing models of adult cortical plasticity to include experience-dependent remodeling of the ECM, highlighting it as a promising target for therapeutic intervention in stress-related disorders.

## Supplementary Information

Below is the link to the electronic supplementary material.

Supplementary Material 1

Supplementary Material 2

## Data Availability

The datasets generated during and/or analyzed during the current study are available from the corresponding author on reasonable request.
