# Supplementary material for: Limited sucrose intake produces subregion-specific remodeling of perineuronal nets in the medial prefrontal cortex
Source: Brain Struct Funct. 2026 Jul 15;231(7):98. doi: 10.1007/s00429-026-03141-5 (PMC13372925; doi:10.1007/s00429-026-03141-5)
Supplement: Supplementary file 1 — Supplementary Material 1 [file 429_2026_3141_MOESM1_ESM.docx]

Limited sucrose intake produces subregion-specific remodeling of perineuronal nets in the medial prefrontal cortex

*Brain Structure and Function*

Houda Nashawi^1,2^, Corey T. Foltz^1^, Nakyung Oh^1^, Eve A. Santiago^1^, Dana R. Selm^1^, James P. Herman^1^, and Yvonne M. Ulrich-Lai^1^

^1^ Department of Pharmacology, Physiology, and Neurobiology, University of Cincinnati, Cincinnati, OH, USA. ^2^ Neuroscience Graduate Program, University of Cincinnati, Cincinnati, OH, USA.

**Corresponding author:**

Yvonne M. Ulrich-Lai, Ph.D.

Professor

Department of Pharmacology, Physiology, and Neurobiology

University of Cincinnati College of Medicine, Reading Campus

2120 E Galbraith Rd – ML 0512

Cincinnati, OH 45237

Phone: 513-558-6118

Email: [yvonne.ulrich-lai@uc.edu](mailto:yvonne.ulrich-lai@uc.edu)


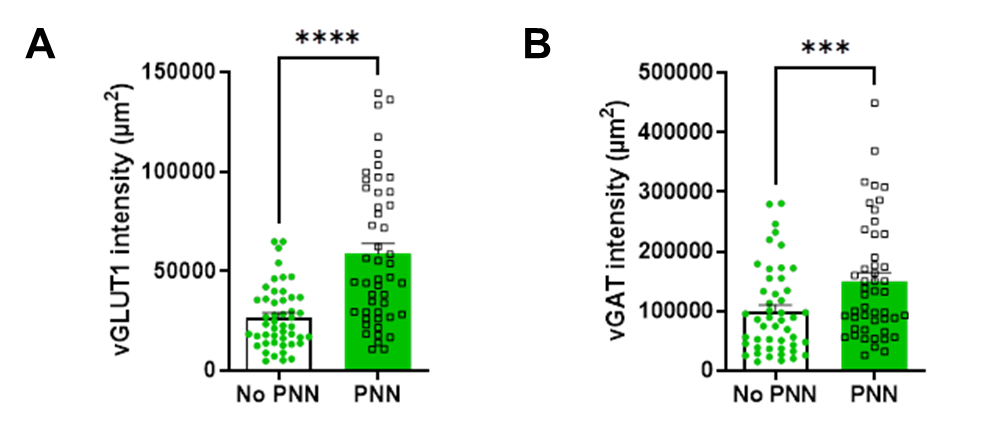
**Effects of perineuronal net (PNN) presence on synaptic terminal labeling intensity in PV+ interneurons of the basolateral amygdala (BLA).** PV+ cells with PNNs exhibited significantly higher vGLUT1 (A) and vGAT (B) labeling intensity compared to PV+ cells without PNNs (p < 0.0001 and p < 0.001, respectively). Data were analyzed with paired t-tests and are presented as mean ± SEM, collapsed across treatment groups. n = 48 rats/group (collapsed across experimental conditions).
