# Supplementary material for: Limited sucrose intake produces subregion-specific remodeling of perineuronal nets in the medial prefrontal cortex
Source: Brain Struct Funct. 2026 Jul 15;231(7):98. doi: 10.1007/s00429-026-03141-5 (PMC13372925; doi:10.1007/s00429-026-03141-5)
Supplement: Supplementary file 2 — Supplementary Material 2 [file 429_2026_3141_MOESM2_ESM.docx]

Limited sucrose intake produces subregion-specific remodeling of perineuronal nets in the medial prefrontal cortex

*Brain Structure and Function*

Houda Nashawi^1,2^, Corey T. Foltz^1^, Nakyung Oh^1^, Eve A. Santiago^1^, Dana R. Selm^1^, James P. Herman^1^, and Yvonne M. Ulrich-Lai^1^

^1^ Department of Pharmacology, Physiology, and Neurobiology, University of Cincinnati, Cincinnati, OH, USA. ^2^ Neuroscience Graduate Program, University of Cincinnati, Cincinnati, OH, USA.

**Corresponding author:**

Yvonne M. Ulrich-Lai, Ph.D.

Professor

Department of Pharmacology, Physiology, and Neurobiology

University of Cincinnati College of Medicine, Reading Campus

2120 E Galbraith Rd – ML 0512

Cincinnati, OH 45237

Phone: 513-558-6118

Email: [yvonne.ulrich-lai@uc.edu](mailto:yvonne.ulrich-lai@uc.edu)

**
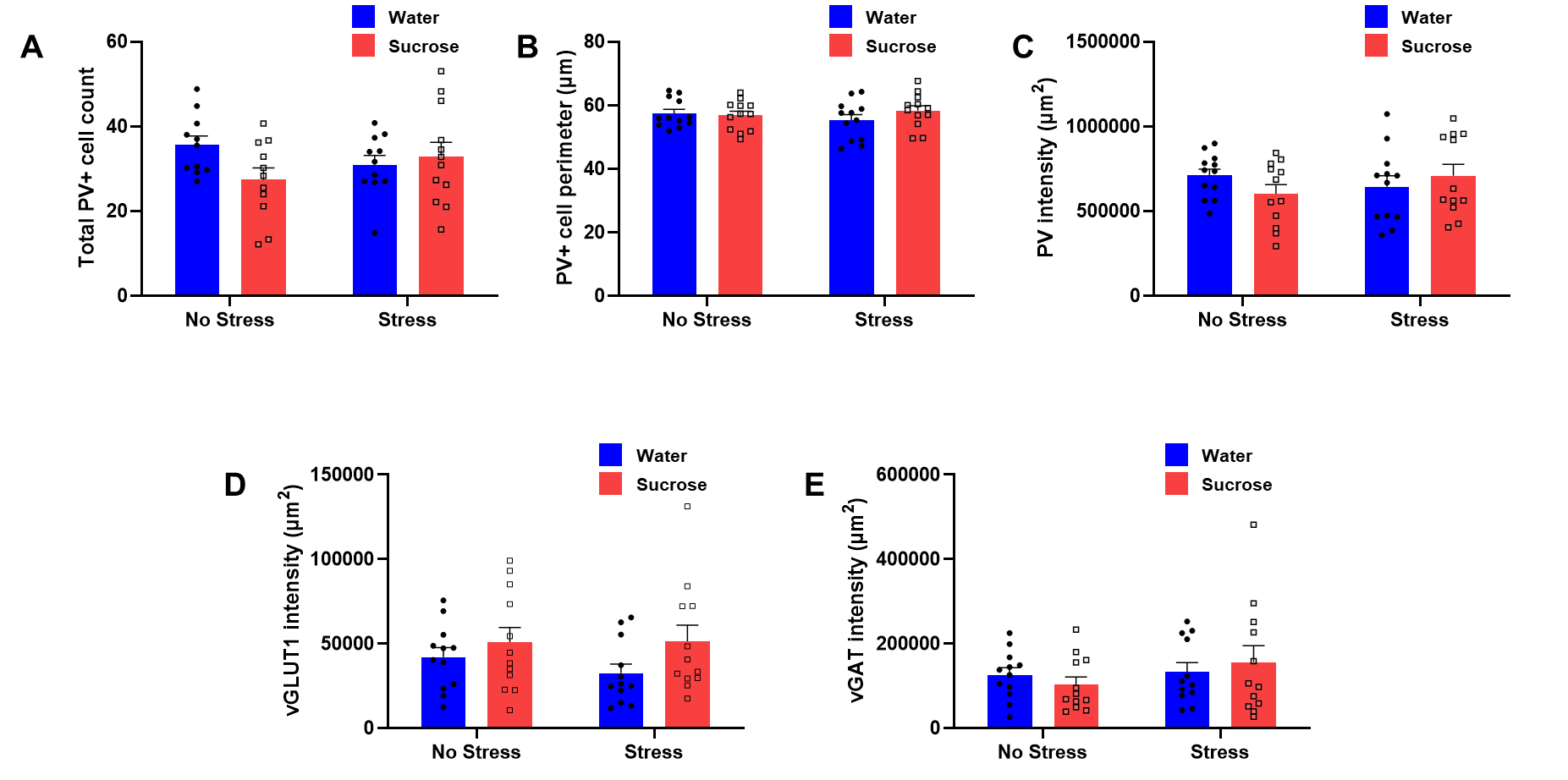
**

**Effects of limited sucrose intake (LSI) and repeated restraint stress on PV+ interneuron properties and synaptic appositions in the basolateral amygdala (BLA).** Neither stress exposure nor sucrose consumption significantly altered PV+ cell count (A), PV+ cell size (B), or PV expression (C) in the BLA. Neither condition influenced vGLUT1 (D) or vGAT (E) labeling intensity in presynaptic puncta apposing PV+ cells. Data were analyzed using two-way ANOVAs and are presented as mean ± SEM. No significant main effects of STRESS, DRINK, or STRESS × DRINK interactions were observed for any parameter. N = 12 rats/group.
